# Evaluation of the anterior and overall tooth ratios in the Saudi population versus Bolton's standards

**DOI:** 10.3389/fped.2023.1237137

**Published:** 2023-08-29

**Authors:** Mohammed Awawdeh, Waad Alsaadi, Faris Awadh B. Alraddadi, Renad Alshunaiber, Jood Alessa, Suliman Alsaeed

**Affiliations:** ^1^ Preventive Dental Science Department, College of Dentistry, King Saud bin Abdulaziz University for Health Sciences (KSAU-HS), Riyadh, Saudi Arabia; ^2^ King Abdullah International Medical Research Center, Ministry of National Guard Health Affairs, Riyadh, Saudi Arabia; ^3^ Dental Services, Ministry of the National Guard- Health Affairs, Riyadh, Saudi Arabia; ^4^ Department of Paediatric Dentistry and Orthodontics, College of Dentistry, King Saud University, Riyadh, Saudi Arabia; ^5^ College of Dentistry, King Saud bin Abdulaziz University for Health Sciences (KSAU-HS), Riyadh, Saudi Arabia; ^6^ Ministry of Health, Riyadh, Saudi Arabia

**Keywords:** orthodontics, Bolton ratio, tooth-size discrepancy, Saudi Arabia, diagnosis

## Abstract

Understanding tooth-size discrepancy is essential in the process of diagnosis of maxillary and mandibular relationship. Due to the high incidence of tooth-size disproportion, Practitioners should consider the high incidence of tooth-size disproportion when planning treatment for their patients, as in many cases, this can be a hindrance to obtaining an ideal result. This study aimed to determine the anterior and overall tooth ratios in the Saudi population and compare them with Bolton's standards. A total of 356 patients were recruited. For the anterior ratio, around 25% of the patients had a ratio equal to Bolton's standards (77.2%). Most subjects (53.7%) had a ratio above 77.2%, and the remaining (20%) had a ratio below 77.2%. The mean amount of anterior mandibular excess was 2.17 ± 2.12 mm, and the mean amount of anterior maxillary excess was 2.16 ± 2.08 mm. For the overall ratio, less than half of the participants (43%) had a ratio equal to Bolton's standards (91.3%). Almost 34% had a ratio above 91.3%, while 23% of the participants had a ratio below 91.3%. The mean amount of overall mandibular excess was 2.54 ± 2.37 mm, and the mean amount of overall maxillary excess was 3.31 ± 3.33 mm. The majority of the study sample had an overall and anterior Bolton ratio that is different from the norms of Bolton's standards, with a tendency for increased overall and anterior ratios. Having specific standards for the Saudi population is important for better clinical assessment and treatment outcomes.

## 1. Introduction

There are many variables that can affect the articulation between upper and lower teeth (1, 2). More than a century ago, Edward Angle proposed his seminal classification of malocclusion (3). As the first formal classification of its kind, it was instrumental in helping the orthodontic community to understand the concept of occlusion and teeth articulation. The classification was divided into class I, Class II-1, class II-2 and class III (3). There was an understanding that class I was the goal of orthodontic treatment or at least the “normal” occlusion. However, there was criticism of Angle's classification from different orthodontic scientists, including Ackerman and Dewy (4–8). The main contention was that if a patient has a class I molar relationship, the occlusion might still exhibit other articulation discrepancies such as crowding, spacing, increased overjet, etc. (9). To overcome this limitation, Andrews developed the famous “Six keys of Occlusion” which includes (1) Class I molar relationship, (2) flat or mild curve of Spee, (3) correct teeth angulation, (4) correct teeth inclination, (5) no rotations, (6) tight interproximal contact (9).

These keys were more representative of ideal and normal occlusion than previous occlusion classifications. However, one key variable was not considered until much later when Bolton highlighted the importance of the tooth size ratio between the upper and lower teeth (10, 11). His assertion was that if all six key requirements of ideal occlusion are met, the articulation between the teeth will not be in harmony if the size of the upper and lower teeth are not proportionally balanced. If the lower teeth are wider than normal or if the upper teeth are narrower than normal, the occlusion of the anterior teeth might exhibit an edge-to-edge occlusion (10, 11). On the other hand, if the lower teeth are narrower than normal or if the upper teeth are wider than normal, the occlusion might exhibit an increased overjet. Hence, the orthodontic community has recognized how essential tooth-size discrepancy is in the process of orthodontic diagnosis, assessment of the maxillary and mandibular relationship, and treatment planning. Tooth-size discrepancy is defined as “a relative excess of tooth structure in an arch in relation to the opposing arch with disparity in individual size of teeth” (12). The Bolton anterior ratio is defined as “the ratios of the mesiodistal widths between the six anterior mandibular teeth and the six anterior maxillary teeth (canine to canine)”, whereas the overall ratio is defined as “the mesiodistal widths between the 12 mandibular teeth and the 12 maxillary teeth (first molar to first molar)” (10, 11).

According to Bolton ratio the anterior ratio should be around 77%, meaning that the width of the lower anterior teeth should be 0.77 of the total width of the upper anterior teeth. The overall ratio should be around 91%, meaning that the width of the lower teeth, first molar to first molar, should be 0.91 of the total width of the opposing upper teeth. In most patients, natural teeth are in harmony when it comes to size. However, 5% of the population has a disparity in the sizes of their teeth (1). Due to the high incidence of tooth-size disproportion, practitioners should consider this when planning treatment for their patients, as in many cases this can be a hindrance to obtaining an ideal result. It was reported that tooth size ratios during orthodontic treatment for various arch length and arch perimeter groups must be carefully examined (13).

Based on the basic six keys of occlusion developed by Andrews in 1972, after evaluation of a selected 120 cast models of ideal occlusions (9), a seventh key of occlusion, “correct tooth size”, was advised by McLaughlin et al. (14). Bolton reported that 29% of patients had a tooth-size discrepancy disproportion (11), while Richardson and Malhotra reported a similar disproportion in 33.7% of their patients (15). Crosby and Alexander found anterior tooth size discrepancy to be prevalent among 22.9% of orthodontic patients (16). A similar result was also reported by Freeman et al. where they found the anterior tooth size discrepancy to be evident in 30.6% of orthodontic patients (17). In 2005, Al-Tamimi and Hashim reported that no significant difference was found in the anterior ratio when they examined Saudi military officers compared to Bolton's anterior and overall ratios (18). However, Alkofide and Hashim reported a significant difference in the anterior ratio between males and females when they examined patients with class III malocclusion (19). Furthermore, they reported a significant difference in all malocclusions cases when compared to Bolton's norms, which was also reported by other studies conducted by Lavelle et al. in England and Ta Ta et al. in Southern China (19, 20). In another study it was revealed that no significant sexual discrepancies in Bolton's anterior ratios or total ratios were reported (21).

In the literature, there are significant differences in the reported tooth-size ratios among various ethnic or racial groups as well as different genders when evaluating tooth-size ratios with different types of malocclusions. This was evident in a study by Ta Ta et al. for southern Chinese children and in Araujo et al.'s study for patients in Brazil (22, 23). Alam et al. recorded similar findings, suggesting that different ethnic groups worldwide have distinct Bolton ratios (24). However, there are other studies that reported no significant difference between different types of malocclusions and the discrepancy in both anterior and overall ratios (16, 25, 26). The interarch tooth-size relationship varies between different populations and these variations in the size of teeth are not systematic. The sample that Bolton studied was not specific in terms of population and sex composition, however, the presence of selection bias is likely (27). Therefore, this study's aim was to determine the anterior and overall tooth ratios in the Saudi population and to compare it with Bolton's standards. Such a study is crucial to guide clinicians in determining precise treatment plan for patients that takes into consideration the discrepancies in the dental ratios. No previous studies have investigated these variable in a similar design.

## 2. Materials and methods

### 2.1. Study design

The present study comprised a cross-sectional study to determine the anterior and overall tooth ratios in the Saudi population, comparing them with Bolton's standards using dental casts for patients at the dental clinics in King Abdulaziz Medical City (KAMC) in Riyadh, Saudi Arabia.

### 2.2. Study subjects

Dental casts with permanent dentition from first molar to first molar, of good quality and with no history of previous orthodontic treatment were included in the study. Patients with tooth agenesis or missing teeth, teeth with anomalous shapes, teeth with large restorations that have mesial or distal over contour interproximal or occlusal wear, or interproximal cavitation due to carious lesions were excluded from the study. Inclusion criteria also included adult Saudi patients with age range below 30 years old to eliminate the risk of tooth wear. The sample included both male and female with all classes of occlusion meeting the inclusion criteria. Patients not meeting the inclusion criteria were excluded from the study. The Bolton analysis was recorded by calibrated operator through measuring the mesiodistal width of all teeth of each cast, excluding the second and third molars. All methods were conducted in accordance with the current version (2013) of the Declaration of Helsinki by the World Medical Association (WMA).

### 2.3. Data collection

Dental casts of patients were selected retrospectively, and those which met the inclusion criteria were reviewed. An Excel sheet was used to record the required data. The measurements were performed using a digital calliper to measure the teeth. The mesiodistal length was obtained by measuring the maximum distance between the mesial and the distal contact points of the tooth on a line parallel to the occlusal plane (28). Each arch was measured twice by a single investigator. Repeatability testing was undertaken by measuring 10% of the samples of the teeth width again after three weeks. The validity of the measurement protocol was assessed using the Intraclass Correlation Coefficient (ICC) and excellent reliability was observed with ICC values between 0.91 and 0.96. Measurements were assessed from the right first molar to the left first molar. If the second measurement differed by more than 0.2 mm from the first measurement, the tooth was measured again. All investigators were trained on measurement criteria and calibration of measuring equipment was carried out in advance. All measurements were taken under natural and neon light. Afterwards, the overall and anterior ratio calculations were taken according to Bolton's Analysis.

### 2.4. Statistical analysis

Data was entered and analyzed using the Statistical Package for Social Sciences (SPSS) version 25 (IBM Corp. Released 2017. IBM SPSS Statistics for Windows, Version 25.0. Armonk, NY: IBM Corp.). Descriptive statistics as frequency distributions, means and percentages were calculated for the variables of the study. Inferential statistics were also calculated. One sample *t*-test, to ascertain whether a population differs significantly from a specific value, was also used to compare the average of the anterior ratio of the study's sample against the known value of Bolton's anterior ratio (77.2%). The same test was also used to compare the mean of our overall ratio with the overall Bolton ratio of 91.3%. A *p*-value equal to or below 0.05 was considered significant.

## 3. Results

**Figure 1:**
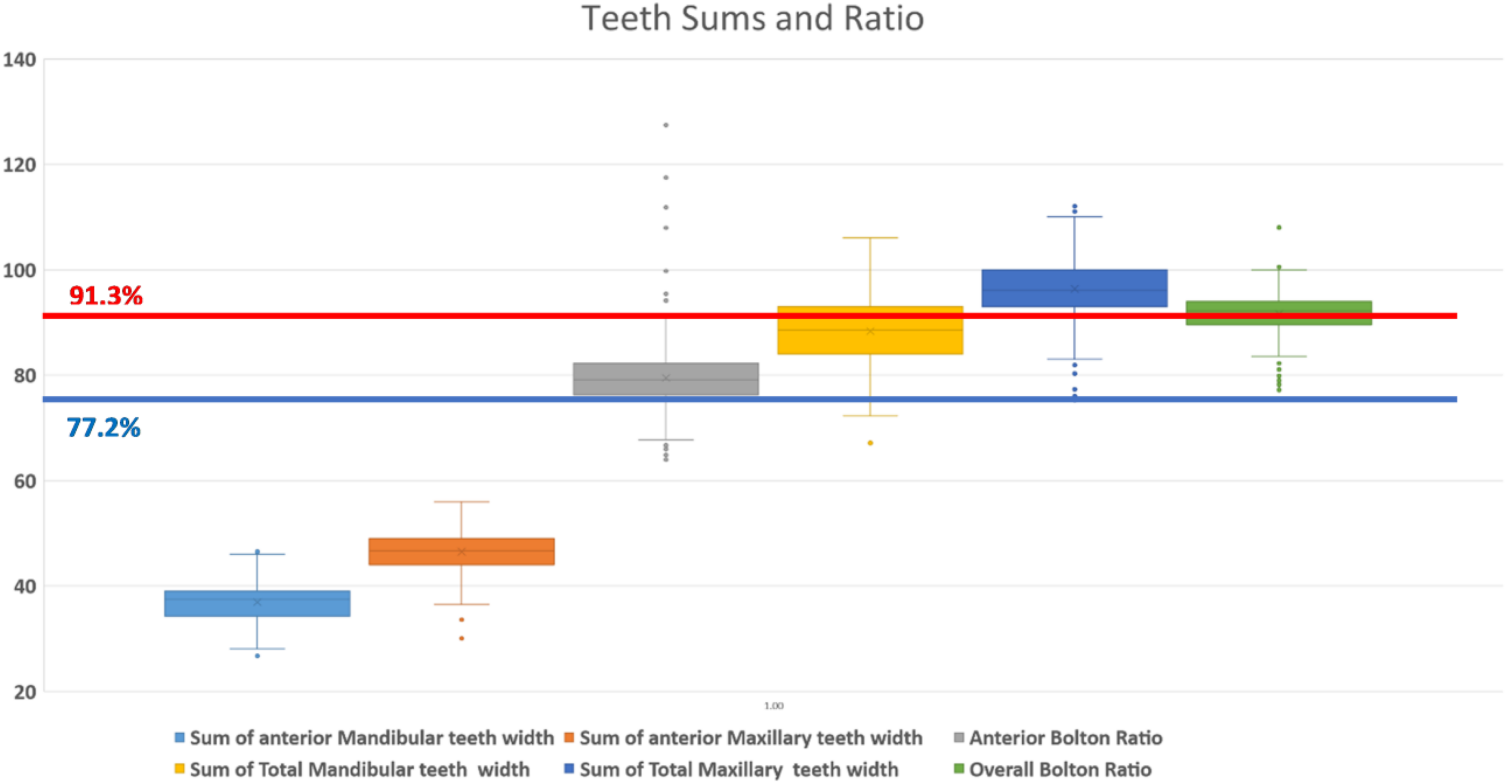
Teeth sums and ratios with Bolton's reference lines.

A total of 356 subjects were included in the study. The mean mesiodistal width value of maxillary anterior teeth was 46.51 ± 3.84 mm and the mean mesiodistal width value of overall maxillary teeth to the distal of the first molar was 96.35 ± 5.83 mm. The mean mesiodistal width value of mandibular anterior teeth was 36.9 ± 3.47 mm and the mean mesiodistal width value of overall mandibular teeth to the distal of the first molar was 88.3 ± 6.20 mm. The mean values of anterior and overall ratios were 79.48 ± 6.95 mm and 91.61 ± 4.08 mm, respectively (see Tables 1, 2, Figure 1).

**Table 1:** Description of the mean and standard deviation for each tooth.

Maxillary teeth	Mean	Standard deviation	Maximum	Minimum
Upper right first molar	10.52	.74	12.00	7.00
Upper right second premolar	7.04	.79	10.00	5.00
Upper right first premolar	7.10	.60	9.00	5.00
Upper right canine	7.73	.78	9.50	4.00
Upper right lateral incisor	6.78	.85	9.00	4.00
Upper right central incisor	8.71	.88	11.00	5.00
Upper left central incisor	8.68	.92	11.00	5.00
Upper left lateral incisor	6.79	.84	9.00	4.00
Upper left canine	7.83	.70	10.00	5.00
Upper left first premolar	7.18	.60	9.00	5.20
Upper left second premolar	7.08	.76	10.00	5.00
Upper left first molar	10.48	.69	12.00	8.00
Mandibular teeth	Mean	Standard deviation	Maximum	Minimum
Lower right first molar	10.80	.82	13.00	9.00
Lower right second premolar	7.34	1.00	11.00	5.00
Lower right first premolar	7.10	.80	11.00	4.00
Lower right canine	6.87	.70	9.00	5.00
Lower right lateral incisor	6.02	.70	8.00	4.00
Lower right central incisor	5.62	.73	10.00	4.00
Lower left central incisor	5.61	.74	10.00	4.00
Lower left lateral incisor	5.95	.72	8.00	4.00
Lower left canine	6.82	.70	9.00	4.00
Lower left first premolar	7.09	.73	9.00	5.00
Lower left second premolar	7.32	.92	11.00	5.00
Lower left first molar	10.80	.87	13.00	8.00

**Table 2:** Descriptive analysis of the teeth sums and ratio.

Variable	Mean	Standard deviation	Variance	Maximum	Minimum	95% confidence interval for mean	
						Lower bound	Upper bound
Sum of anterior maxillary teeth width	46.52	3.84	14.76	56.00	30.00	46.12	46.92
Sum of total maxillary teeth width	96.35	5.84	34.09	112.00	75.30	95.75	96.96
Sum of anterior mandibular teeth width	36.91	3.48	12.10	46.90	26.70	36.55	37.27
Sum of total mandibular teeth width	88.30	6.20	38.45	106.00	67.10	87.65	88.94
Anterior Bolton ratio	79.48	6.95	48.35	127.40	63.97	78.76	80.21
Overall Bolton ratio	91.61	4.09	16.73	108.00	77.19	91.19	92.04

Comparing these findings to the Bolton analysis, the anterior ratio for 26.4% of subjects was equal to Bolton's standards (77.2%). Most subjects (53.7%) had an increased anterior Bolton ratio, while the remaining (19.9%) had a decreased anterior Bolton ratio. The mean amount of anterior mandibular excess was 2.17 ± 2.12 mm, and the mean amount of anterior maxillary excess was 2.16 ± 2.08 mm (see Table 3).

**Table 3:** Description of the anterior Bolton ratio.

Anterior Bolton ratio assessment	Frequency (*n*)	Percent %
Reduced anterior Bolton ratio (Less than 75.55%)	71	19.9%
Normal anterior Bolton ratio (77.2% ± 1.65)	94	26.4%
Increased anterior Bolton ratio (More than 78.85%)	191	53.7%
Total	356	100.0%

For the overall ratio, 43.3% of participants had a ratio equal to Bolton's standards (91.3%). Around 43% had an increased overall Bolton ratio, while 22.8% had a decreased overall Bolton ratio. The mean amount of overall mandibular excess was 2.54 ± 2.37 mm, and the mean amount of overall maxillary excess was 3.31 ± 3.33 mm (see Table 4). Confidence intervals were calculated at 95% using SPSS version 26. The confidence interval was 91.19–92.04 for the overall Bolton and 78.76–80.21 for the anterior Bolton Ratio (see Table 4).

**Table 4:** Description of the overall Bolton ratio.

Overall Bolton ratio assessment	Frequency (*n*)	Percent %
Reduced overall Bolton ratio (less than 89.39%)	81	22.8%
Normal overall Bolton ratio (91.3% ± 1.91)	154	43.3%
Increased overall Bolton ratio (more than 93.21%)	121	34.0%
Total	356	100.0%

One sample *t*-test showed that the anterior ratio (*m* = 79.48 and SD = 91.61) differed significantly (*p* < .001) from the anterior Bolton ratio (%77.2). While the overall ratio (*m* = 91.61, SD = 4.09) did not differ significantly (*p* = 0.15) from the established overall Bolton ratio of 91.3% (see Table 5).

**Table 5:** One sample *t*-test.

	Mean	Std. deviation	Std. error mean	Test value	*t*	df	Sig. (2-tailed)	Mean difference	95% confidence interval of the difference	
									Lower	Upper
Anterior Bolton ratio	79.48	6.95	0.37	77.20	6.19	355.00	0.00	2.28	1.56	3.01
Overall Bolton ratio	91.61	4.09	0.22	91.30	1.45	355.00	0.15	0.31	−0.11	0.74

## 4. Discussion

In order to compare tooth discrepancy in the Saudi population with Bolton's standards which consisted of only Caucasian population, 356 casts were obtained and the mesio-distal width of teeth was measured from the first molar to the first molar in both arches. The means and the standards deviations for both anterior and overall measurements, were larger than those reported in Bolton's standards. This was in agreement with what was reported by Paredes et al. for the Spanish Population (28), Bernabé et al. for the Peruvian Adolescents population (29), and Santoro et al. for the Dominican Americans populations (30). The reason for this finding could be due to the difference in the sample size between this study and Bolton's as well as the difference in the ethnic group. For the anterior ratio, the majority of the population studied (53.7%) had an increased anterior Bolton ratio. This is similar to the findings of Santoro et al. (30), as well as Araujo and Souki for the Brazilian population (23), where they also reported a larger anterior ratio as compared to Bolton's standards. Furthermore, in 2017, Hashim et al. reported that the Qatari population, which is closer to the Saudi population, also reported a statistical significance when the anterior ratio was compared to that of Bolton (31). In 2014, Subbarao et al. also reported similar findings on the Indian population where both anterior and overall ratios of Bolton did not apply (32). On the other hand, in 2003, Alkofide and Hashim reported that there was no difference in the anterior ratio of their population as compared to that of Bolton (19). Moreover, Al-Tamimi and Hashim published similar results in 2005 on the Saudi population (18). Furthermore, when studying the applicability of Bolton's analysis on the Japanese population, Endo et al. found no statistical significance in both anterior and overall ratios to Bolton's standards (25). Most participants had an increased overall Bolton ratio, which means in our populations there is, predominantly, increased mandibular excess, which is contrary to the result found by Santoro et al. (30).

Recently, a systematic review and meta-analysis aiming to estimate the tooth size discrepancy values for the Saudi population was conducted (33). The study was done by analyzing the data from eight studies on the Saudi population (33). The reported values were 79.08 ± 3.4 for the anterior ratio for all occlusal relationships (Class I, II or III) and for both genders (33). For the overall ratio, the study suggested a value of 92.51 ± 2.82, except for class III cases where the value was set at 91.97 ± 2.4 for females and 93.13 ± 2.6 for males (33). It was noted that most of the studies included in this systematic review had a relatively small sample size compared to this study where we evaluated 356 cases (34–37). However, the finding from the systematic review was not dissimilar from the results of this study, especially for the anterior ratio where both studies agreed on a value of 79%. For the overall ratio, there was a small, not clinically significant, difference between both studies (92.5 vs. 91.61). Moreover, the result of this study was similar to the studies done with Qatari and Japanese populations, exhibiting no statistical difference, but ratios higher than those suggested by Bolton (31).

The discrepancy between the size of the upper and lower teeth can be managed clinically by different techniques (1, 38, 39). Firstly, the nature of the discrepancy has to be determined to establish whether it is an increased or decreased anterior or overall ratio. Then, the cause of this discrepancy needs to be determined. For instance, if a patient has a significantly increased anterior ratio (e.g., 80%), both the upper and lower anterior teeth need to be examined closely to evaluate if the increase in this ratio is due to wide lower anterior teeth or narrow upper anterior teeth. Hence, supporting data, such as the Golden Proportion and the reported normal widths of upper and lower incisors, can be used to conclude the cause of this increased anterior ratio. It is not uncommon to see cases with narrow upper lateral incisors which can result in an increased anterior ratio, hence the ideal plan should include composite build ups, veneers or crowns to restore the width of the upper teeth to reach a normal anterior ratio (40, 41). However, if the upper incisors are normal in width, an interproximal reduction can be performed on the lower anterior teeth to achieve harmony in occlusion with positive overjet (1, 38, 39).

In this study, the gender was not specified. Although this might be considered a limitation, most of the studies on the tooth size discrepancy could not find a significant difference between males and females, and if it exists, it was mostly not clinically or statistically significant (42–45). However, there are studies that proposed the idea that females might have narrower teeth than males, but this will be generalized to both the upper and lower teeth, which will keep the ratio unaffected (46–48).

Another potential limitation of this study was that the occlusion type was not studied. However, most of the studies on tooth size discrepancy have not found a difference in Bolton's ratio between class I, II or III cases (26, 49–51). This can be explained by the fact that Angle's classification evaluated the malocclusion in the anteroposterior plane which is mostly affected by the position of the jaws or the drifting of the teeth and not the tooth size proportion (3, 10).

## 5. Conclusions

The majority of the study sample had an overall and anterior Bolton ratio that is different from the norms of Bolton's standards, with a tendency for increased overall and anterior ratios. Having specific standards for the Saudi population is important for better clinical assessment and treatment outcomes. Overall, it is recommended to conduct more research on the Saudi population to confirm the findings of the Bolton's discrepancy outlined in this study.

## Data Availability

The raw data supporting the conclusions of this article will be made available by the authors, without undue reservation.
